# Thrombospondin-2 promotes prostate cancer bone metastasis by the up-regulation of matrix metalloproteinase-2 through down-regulating miR-376c expression

**DOI:** 10.1186/s13045-017-0390-6

**Published:** 2017-01-25

**Authors:** Po-Chun Chen, Chih-Hsin Tang, Liang-Wei Lin, Chun-Hao Tsai, Cheng-Ying Chu, Tien-Huang Lin, Yuan-Li Huang

**Affiliations:** 1 Graduate Institute of Basic Medical Science, China Medical University, Taichung, Taiwan; 2 Department of Pharmacology, China Medical University, Taichung, Taiwan; 3 Department of Biotechnology, College of Medical and Health Science, Asia University, Taichung, Taiwan; 4 The Ph.D. Program for Cancer Biology and Drug Discovery, Taipei Medical University, Taipei, Taiwan; 5 Department of Urology, Buddhist Tzu Chi General Hospital Taichung Branch, Taichung, Taiwan; 6 Department of Orthopedic Surgery, China Medical University Hospital, China Medical University, Taichung, Taiwan; 7 Department of Medical Research, China Medical University Hospital, China Medical University, Taichung, Taiwan

**Keywords:** Thrombospondin-2, Metastasis, Prostate cancer, microRNA, Matrix metalloproteinase-2

## Abstract

**Background:**

Thrombospondin-2 (TSP-2) is a secreted matricellular glycoprotein that is found to mediate cell-to-extracellular matrix attachment and participates in many physiological and pathological processes. The expression profile of TSP-2 on tumors is controversial, and it up-regulates in some cancers, whereas it down-regulates in others, suggesting that the functional role of TSP-2 on tumors is still uncertain.

**Methods:**

The expression of TSP-2 on prostate cancer progression was determined in the tissue array by the immunohistochemistry. The molecular mechanism of TSP-2 on prostate cancer (PCa) metastasis was investigated through pharmaceutical inhibitors, siRNAs, and miRNAs analyses. The role of TSP-2 on PCa metastasis in vivo was verified through xenograft in vivo imaging system.

**Results:**

Based on the gene expression omnibus database and immunohistochemistry, we found that TSP-2 increased with the progression of PCa, especially in metastatic PCa and is correlated with the matrix metalloproteinase-2 (MMP-2) expression. Additionally, through binding to CD36 and integrin α_ν_β_3_, TSP-2 increased cell migration and MMP-2 expression. With inhibition of p38, ERK, and JNK, the TSP-2-induced cell migration and MMP-2 expression were abolished, indicating that the TSP-2’s effect on PCa is MAPK dependent. Moreover, the microRNA-376c (miR-376c) was significantly decreased by the TSP-2 treatment. Furthermore, the TSP-2-induced MMP-2 expression and the subsequent cell motility were suppressed upon miR-376c mimic stimulation. On the other hand, the animal studies revealed that the bone metastasis was abolished when TSP-2 was stably knocked down in PCa cells.

**Conclusions:**

Taken together, our results indicate that TSP-2 enhances the migration of PCa cells by increasing MMP-2 expression through down-regulation of miR-376c expression. Therefore, TSP-2 may represent a promising new target for treating PCa.

**Electronic supplementary material:**

The online version of this article (doi:10.1186/s13045-017-0390-6) contains supplementary material, which is available to authorized users.

## Background

Prostate cancer (PCa) is one of the most frequently diagnosed cancers in men and is the second leading cause of cancer deaths for men in the USA [1]. The majority of deaths are due to the failures of diagnosis, current therapies, and subsequent development of the metastatic cancer, 80% of which is primarily metastasize to the bone [2]. Thus, better strategies for the treatment of PCa will ultimately require a better understanding of the molecular mechanisms that trigger and drive cancer progression and metastatic processes.

Metastasis is a process that tumor cells leave a primary location, travel through circulation, and form a secondary tumor in the distant organ. Bone metastasis is a common complication associated with advanced cancers, including PCa, often causing acute pain and bone fractures, and the major reason for the cause of deaths in patients [3]. The breakdown of the basement membrane surrounding the tumors as well as the increase in the abilities of proliferation, migration, and invasion are the hallmarks of metastasis [4]. The well-studied protease linked with migration and invasion is the matrix metalloproteases (MMPs). MMP-2 and MMP-9, also known as gelatinases, play a key role for cleaving type I, IV collagen and contribute to the process of metastasis [5]. In PCa, MMP-2 and MMP-9 are considered useful prognostic markers and are correlated to PCa progression [6], indicating that the interaction between tumor cells and extracellular matrix is associated with tumorigenesis. Thus, targeting the factors that can regulate the activity of MMPs may develop as a strategy to suppress PCa progression.

Thrombospondin (TSP), a glycoprotein that belongs to a group of matricellular proteins, participates in cell-to-cell and cell-to-matrix communication [7]. The human TSP protein family consists of five members, named TSP-1~5 [8]. TSP-1 and TSP-2 are highly expressed during the tissue remodeling that is associated with cancer progression, whereas the roles of the other members of TSPs in tissue remodeling are less well understood. The structure of TSP-1 and TSP-2 is similar, but their expression patterns are temporally and spatially different during mouse development, suggesting that they may play different roles [7–9]. In response to injury, the expression of TSP-2 is increased and is associated with tumor growth [8]. In addition, TSP-2 might play a role in collagen fibrillogenesis in the skin and tendons, suggesting that TSP-2 modulates the cell surface properties of mesenchymal cells, and thus, regulates cell functions, such as adhesion and migration [10]. Through binding to CD36 with their type I repeats, TSP-1 and TSP-2 have been reported to serve as an anti-angiogenic molecule [11]. Additionally, the arginine-glycine-aspartic acid (RGD) sequence in TSP-2 has been reported that it binds to integrin α_ν_β_3_ and heparan sulfate proteoglycans which are associated with cell adhesion or binds to the low-density lipoprotein receptor-related protein (LRP) that modulates the concentration of TSP-2 in the pericellular environment by endocytosis and lysosomal degradation of the protein [12]. Through interaction with LRP, TSP-2 binds to pro-MMP-2 and MMP-2 that regulates the extracellular levels of MMP-2 which is important for controlling several physiological processes, such as collagen fibrillogenesis, wound healing, and angiogenesis [13, 14]. Moreover, TSP-1, TSP-2 was also demonstrated to play roles on anti-angiogenesis in the tumors [11, 15]. However, the expression profile of TSP-2 is quite controversial, which is down-regulated in cervical cancer [9] and ovarian cancer [16], while it is overexpressed in oral cavity squamous cell carcinoma [17], pulmonary adenocarcinoma [18], and prostate cancer [19], suggesting that TSP-2 may play another role rather than anti-angiogenesis. These studies indicate that the role of TSP-2 on tumorigenesis is still controversial, especially on metastasis.

MicroRNAs (miRNAs) are small non-coding RNA that regulates gene expression through binding to 3′ untranslated region (3′UTR) of mRNA, which with important functions in development, cell differentiation, regulation of cell cycle, and apoptosis [20, 21]. Through up- or down-regulation of tumor suppressor genes, miRNAs may function in either an oncogenic or tumor suppressor role and they appear to play important and unique roles with respect to PCa progression [22]. Several miRNAs have been illustrated to mediate metastasis, including miR-154, miR-376c, miR-377, miR-381, and miR-495 [23]. It has been shown that miR-376c enhances proliferation, survival, and chemo-resistance by targeting activin receptor-like kinase 7 in ovarian cancer [24]. Whereas, others showed that miR-376c inhibit cell proliferation and invasion by targeting the transforming growth factor-α in osteosarcoma [25]. Thus, miR-376c may play “dual” roles on tumor progression. However, its role on PCa cells is largely uncertain. Herein, we showed for the first time that TSP-2 up-regulates MMP-2 expression and the subsequent cell motility in human PCa cells. Furthermore, these TSP-2’s effects are dependent on down-regulation of miR-376c expression.

## Methods

### Reagents

Recombinant human TSP-2 was purchased from R&D Systems (Minneapolis, MN, USA). The anti-rabbit TSP-2 was obtained from Abnova (Taipei, Taiwan); anti-rabbit p38, anti-mouse p-ERK, ERK, p-JNK, and p-p38 were obtained from Santa Cruz (CA, USA); anti-mouse MMP-2 was from R&D Systems (MN, USA). The inhibitors for p38 (SB203580), ERK (U0126), JNK (SP600125), and MMP-2 (MMP-2 inhibitor I) were purchased from Calbiochem (San Diego, CA, USA). The inhibitors for integrin α_4_β_1_ (sulfo-*N*-succinimidyl oleate (SSO)), CD36 (BIO1221), integrin α_v_β_3_ (RGD), and the control peptide (RAD) were purchased from Torcis Bioscience (Ellisville, MO, USA). The Luciferase assay kit was purchased from Promega (Madison, WI, USA). TSP-2 shRNA plasmids were purchased from National RNAi Core Facility Platform (Taipei, Taiwan). The TSP-2 shRNA oligo sequences were 5′-CCGGCCCTCCTAAGACAAGGAACATCTCGAGATGTTCCTTGTCTTAGGAGGGTTTTTG-3′ which target to the sequence of TSP-2 is 5′-CCCTCCTAAGACAAGGAACAT-3′. MiR-376c mimic was purchased from Invitrogen (Carlsbad, CA, USA). All other chemicals were purchased from Sigma-Aldrich (St. Louis, MO, USA).

### Microarray data

The TSP-2 gene expression profile data was retrieved from the GEO database (http://www.ncbi.nlm.nih.gov/geo/). A total of 182 normal prostate tissues, as well as benign, primary, and metastatic prostate tumors were obtained from the microarray data with the following accession numbers: GDS1439 and GDS2545. The TSP-2 gene expression profiles were retrieved from GEO data analysis tools. Further analysis of TSP-2 gene expression between normal, primary tumor, and metastatic tumor were examined by one-way ANOVA with Bonferroni’s multiple comparisons test and *p* value ≤0.05 showed significance.

### Cell culture

Human PCa cell lines (PC-3 and DU145) and human normal prostate epithelial cell lines (PZ-HPV7) were obtained from the American Type Culture Collection (ATCC). PC-3 and DU145 cells were grown in RPMI-1640 medium supplemented with 20 mM HEPES, 10% heat-inactivated fetal calf serum, 2 mM glutamine, 100 U/ml penicillin, and 100 μg/ml streptomycin. PZ-HPV7 cells were grown in keratinocyte-SFM, containing bovine pituitary extract and recombinant epidermal growth factor. All cells were maintained in a humidified incubator at 37 °C, 5% CO_2_.

### Transwell assay

The migration assay was performed using the transwell plates (Costar, NY, USA). The invasion assay was performed using the same transwell plate except for coating Corning® Matrigel® Matrix (Corning, NY, USA) in the lower chamber. Selection of invasive prostate cancer cells (PC-3-I3 and DU145-I5) was performed by using transwell invasion assay as described previously [26]. Briefly, the transwell inserts were coated with Matrigel, prostate cancer cells were resuspended in 1% FBS containing media and seeded into the wells in the upper layer, with the lower layer supplied with 10% FBS containing media. After 48 h, the inserts were removed, and cells that had migrated through the membranes and become attached to the lower chamber compartments were trypsinized and expanded for second-round selection. The PC-3-I3 and DU145-I5 invasive prostate cancer cells were established after 3 and 5 rounds of selection, respectively.

### Small interfering RNA (siRNA) transfection

Cells were transfected with siRNAs according to manufacturers’ recommendations on standard procedure [26, 27]. The siRNAs (ON-TARGETplus SMARTpool) were purchased from GE Dharmacon (Lafayette, CO, USA). Cells were transfected with siRNA using Lipofectamine 2000 reagent. The mRNA knockdown efficiency was confirmed by real-time PCR as described in the following sections.

### Reverse transcription (RT) and real-time PCR

Total RNA was extracted from PCa cells using a TRIzol kit as described previously [26]. Briefly, the reverse transcription reaction was performed using the oligo (dT) primer. Real-time PCR analysis was carried out using SYBR with sequence-specific primers. The GAPDH mRNA expression was used as an internal control.

For miRNA detection, reverse transcription was performed using Mir-X™ miRNA First-Strand Synthesis and SYBR® RT-PCR with the specific forward primer of miR-376c (5′-AACATAGAGGAAATTCCACGT-3′). The U6 snRNA was used for normalization. The threshold was set above the non-template control background and within the linear phase of target gene amplification to calculate the cycle number at which the transcript was detected (denoted as CT).

### Immunoblotting assay

Protein was isolated from PCa cells, and its concentration was then determined as described previously [27]. Proteins were resolved by SDS-PAGE and transferred to Immobilon polyvinylidene fluoride membranes. After incubation with primary and secondary antibodies, the membranes were visualized by enhanced chemiluminescence using Kodak X-OMAT LS film.

### Zymography analysis

The supernatants of the indicated condition of cells were mixed with sample buffer without reducing agent and heating. The sample was performed with SDS-PAGE containing gelatin (1 mg/ml). Afterwards, the gel was washed with 2.5% Triton X-100 to remove SDS, rinsed with 50 mM Tris–HCl, pH 7.5, and then incubated overnight at room temperature with the developing buffer (50 mM Tris–HCl, pH 7.5, 5 mM CaCl_2_, 1 μM ZnCl_2_, 0.02% thimerosal, 1% Triton X-100). The zymographic activities were revealed by staining with 1% Coomassie blue. The same samples were performed with SDS-PAGE without gelatin and staining with 1% Coomassie blue as loading control.

### Immunohistochemical (IHC) staining

The protein expression was determined on tissue slides using IHC staining as described previously [26]. Human PCa tissue array (T195b and PR956) was purchased from Biomax (MD, USA) in the form of 5 μm sections of paraffin-embedded tissue on glass slides. The tissue slides were incubated with human TSP-2 and MMP-2 antibodies, followed by counterstaining with hematoxylin.

### Plasmid construction and luciferase activity assay

The 3′UTRs of the human MMP-2 gene were amplified by PCR using the following primer: Forward primer, 5′-GAGTTTAAACCCTCTTTAAGTCTGTTTCTTC-3′, Reverse primer, 5′-GCGCTAGCCAACTAATAATGGCCTTTTT-3′. The 3′UTRs of MMP-2 were cloned downstream of the reporter gene in the pGL2-Control vector. The predicted MMP-2 binding site for miRNA was identified by the miRDB (http://mirdb.org/miRDB). Mutant plasmids that attenuate the interaction between MMP-2 3′UTR and miRNA were generated using a QuikChange Site-Directed Mutagenesis kit (Stratagene, Cedar Creek, TX, USA). Mutagenesis of miR-376c targeting seed region of MMP-2 3′UTR were amplified by PCR using the following primers: Forward primer, 5′-CAATTAATAGAGTGCTTTCTGGGTGCAAGGCACTTTTCACG-3, Reverse primer, 5′-CGTGAAAAGTGCCTTGCACCCAGAAAGCACTCTATTAATTG′-3”. These plasmids with 3′UTR of MMP-2 and β-galactosidase as control were transfected into cells using lipofectamine 2000. Following transfection, these cells were incubated with the indicated agents. Cell extracts were prepared and used for measuring the luciferase and β-galactosidase activities as the manufacturer’s recommendations. Activities of luciferase and β-galactosidase were then measured by Luciferase Assay System (Promega, WI, USA).

### In vivo metastasis model

PC-3 constitutively expressed pLenti CMV V5-Luc cells were transfected with TSP-2 shRNA plasmids and selected by puromycin. PC-3 stable cells (2 × 10^6^) which containing control vector or TSP-2 shRNA-expressing vector were intratibially injected with 200 μl Matrigel into the right tibia of nude mice (4-week old). Bone metastasis was monitored using an in vivo imaging system (Xenogen IVIS imaging system). The mice were humanely sacrificed after 4 weeks. The tumor mass was removed from the right tibia bone to detect its weight.

### Microcomputed tomography (micro-CT) analysis

After the mice were sacrificed, the tibia of tumor growth were dissected and fixed in 4% paraformaldehyde for micro-CT analysis. The tibia for micro-CT scanning was assessed by using a micro-CT scanner (Skyscan 1176; Bruker, Kontich, Belgium). Reconstruction of sections was carried out with GPU-based scanner software (NRecon, Bruker). In addition, the grayscale was based on the Hounsfield unit, and the validated calcium standards were scanned as the density reference. The three-dimensional microstructural volumes from the micro-CT scans were analyzed using Skyscan software (CTAn, Bruker). Bone volume was used to assess the bone resorption area of the bone metastasis.

### Meta-analyses of microarray datasets from The Cancer Genome Atlas (TCGA) database

The 499 prostate adenocarcinoma datasets were retrieved from the TCGA. The datasets included mRNA, microRNA expression levels, and clinical data. Total 50 paired adjacent normal and tumor specimens were used to analyze expression level of TSP-2 and miR-376c. The paired *t* test was performed, and *p* value ≤0.05 showed significance. The TSP-2 and MMP-2 expression levels were correlated with Gleason score of the tumor specimens. The one-way ANOVA with Bonferroni’s multiple comparisons test was performed, and *p* value ≤0.05 showed significance. The correlation between TSP-2 and MMP-2 was analyzed by all mRNA sequencing results in the dataset.

### Statistical analysis

All data presented as mean ± standard error of the mean (SEM). Statistical analysis between the two samples was performed using the Student *t* test. *p* < 0.05 was considered significant.

## Results

### The expression of the TSP-2 is correlated with PCa progression

**Fig. 1:**
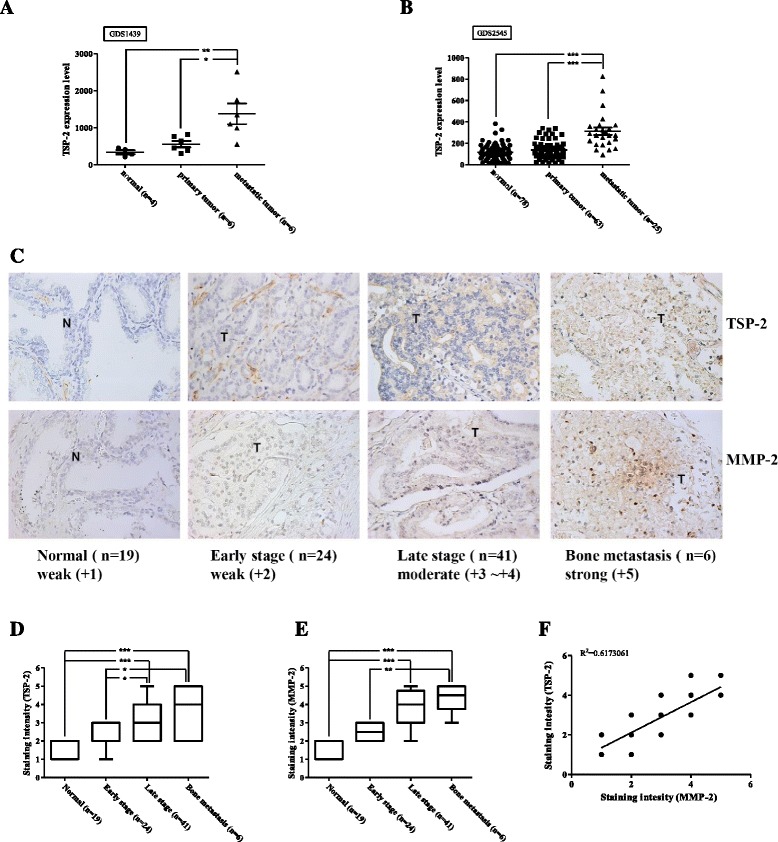
The expression of TSP-2 is correlated with MMP-2 expression and tumor stage in PCa. The *THBS2* genes were searched from the GEO database. **a** GDS1439 and **b** GDS2545 were detected by microarray, and the TSP-2 expression values were collected independently. **c** TSP-2 and MMP-2 protein were determined in the tissue array by IHC staining using their specific antibodies. The sections were photographed, and staining intensity was then scored from 0 to 5 based on their expression intensity. The expression of TSP-2 (**d**) and MMP-2 (**e**) were quantified by the clinical pathologic stages (stage I and II for early stage, stage III and IV for late stage), and **f** the correlation between TSP-2 and MMP-2 were analyzed with Pearson correlation. The one-way ANOVA with Bonferroni’s multiple comparisons test was performed and *p* ≤ 0.05 showed significance. *N* normal, *T* tumor

**Fig. 2:**
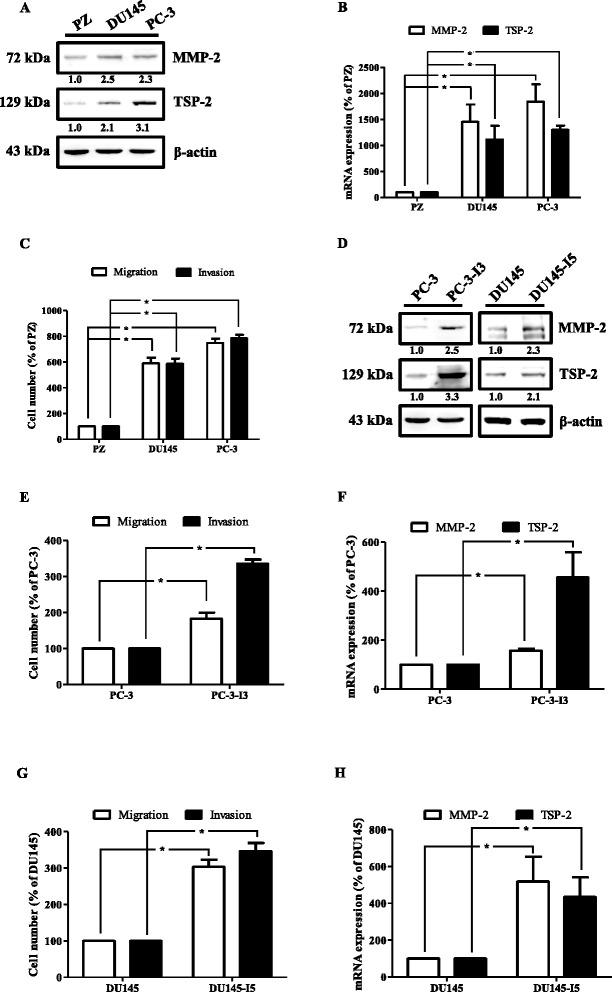
TSP-2 is highly expressed in metastatic human PCa cells. **a** Total proteins were extracted from PZ-HPV7, DU145, and PC-3 cells and subjected to immunoblotting analysis using anti-MMP-2 and TSP-2 antibodies, and re-probed against β-actin to show equal loading amounts. **b** The MMP-2 (*open bar*) and TSP-2 (*closed bar*) mRNA expressions of PZ-HPV7, DU145, and PC-3 cells were examined by real-time PCR. **c** The migratory (*open bar*) and invasive (*closed bar*) abilities of PZ-HPV7, DU145, and PC-3 cells were determined by the transwell assay. **d** Total protein was extracted from PC-3, PC-3-I3, DU145, and DU145-I5 cells and subjected to immunoblotting analysis using anti-MMP-2 and TSP-2 antibodies. The MMP-2 and TSP-2 mRNA expression (**f** and **h**) as well as migration and invasion (**e** and **g**) ability of PC-3, PC-3-I3, DU145, and DU145-I5 cells were determined by RT-PCR and the transwell assay, respectively. Results are expressed as mean ± SEM. *p < 0.05 when compared to PZ-HPV-7, PC-3, or DU145 cells

The previous studies of TSP-2 in tumor progression are still controversial. To examine the correlation and role of TSP-2 in PCa progression, we therefore manipulated the GEO database. The two independent datasets of PCa were retrieved and analyzed, and we found that the expression of the TSP-2 gene was higher in PCa, especially on metastatic tumors (Fig. 1a, b). Surprisingly, these results were not consistent with the previous investigation [19]. To further confirm the TSP-2 expression levels on PCa progression in vivo, the samples from the different stages of PCa patients were collected. Enormous studies have indicated the crucial roles of MMP-2 in PCa prognostic significance progression [28, 29]. Therefore, we assessed the correlation between TSP-2 and MMP-2 expression in PCa specimens. The expressions of TSP-2 and MMP-2 in patients with PCa were higher than that in normal individuals (Fig. 1c–e). Additionally, the expressions of TSP-2 and MMP-2 were positively correlated and highly expressed in prostate-bone metastatic compared to the localized tumor (Fig. 1d, e), indicating that TSP-2 was associated with clinical pathologic stages in PCa. Furthermore, the expression of MMP-2 was positively correlated to TSP-2 (Fig. 1f). Meanwhile, we also showed that the expressions of TSP-2 and MMP-2 as well as the migratory and invasive abilities were higher in two human PCa cell lines, PC-3 and DU145, than that in normal prostate epithelial PZ-HPV7 cells (Fig. 2a–c). MMP-2 is an established pro-migratory protease that participates in cancer migration and invasion, suggesting that TSP-2 regulates MMP-2 followed by modulating migration and invasion in PCa cells. To clarify this issue, highly migratory PC-3-I3 and DU145-I5 cells were selected by transwell assay (Fig. 2d, e, and g). These cells also showed higher MMP-2 and TSP-2 expressions than the PC-3 and DU145 cells (Fig. 2f, h). Taken together, these results indicated that the expression of TSP-2 is associated with cell migration, invasion, and PCa progression.

### MMP-2 is involved in TSP-2-induced cell migration and invasion in human PCa cells

**Fig. 3:**
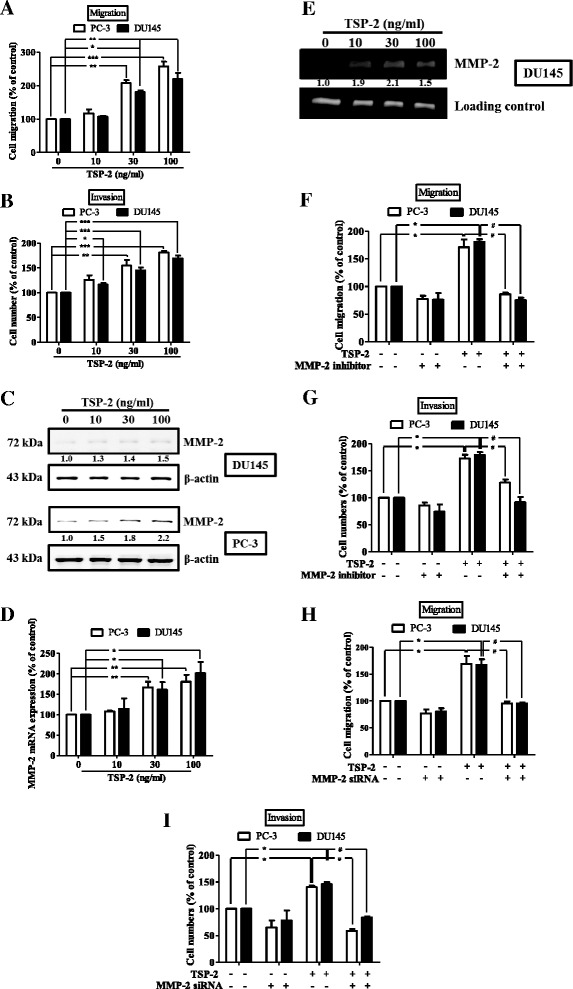
TSP-2 induces migration and invasion in human PCa cells. PC-3 (*open bar*) and DU145 (*closed bar*) cells were incubation with TSP-2 (10–100 ng/ml) for 24 h, and their abilities of migration (**a**) and invasion (**b**) were measured by the transwell assay, and the MMP-2 protein and mRNA expressions as well as its activity were analyzed through immunoblotting (**c**), real-time PCR (**d**), and zymography (**e**) analyses, respectively. PC-3 (*open bar*) and DU145 (*closed bar*) cells were pretreated with vehicle control (DMSO) or 2 μM of MMP-2 inhibitor (**f** and **g**) for 30 min or transfected with MMP-2 siRNA (**h** and **i**), followed by stimulation with or without TSP-2 for another 24 h, and then assessed their migratory (**f** and **h**) and invasive (**g** and **i**) abilities. Cells without treatments were used as a control (set to 100), and data were shown as multiples of that. Results are shown as the mean ± SEM (*n ≥* 3). The one-way ANOVA with Bonferroni’s multiple comparisons test was performed, and *p* ≤ 0.05 showed significance. *p < 0.05 when compared to untreated control; ^#^ *p* < 0.05 when compared to the TSP-2-treated group

To verify the migratory effect of TSP-2 on PCa cells, PC-3 and DU145 cells were treated with various concentrations of TSP-2. Results showed that both migratory and invasive activities significantly increased upon TSP-2 treatment via a concentration-dependent manner (Fig. 3a, b, and Additional file 1: Figure S3A). Meanwhile, the expressions of MMP-2 protein and mRNA as well as the amount of active MMP-2 were also enhanced by TSP-2 stimulation (Fig. 3c–e and Additional file 1: Figure S1A and B). To clarify whether MMP-2 is involved in the TSP-2-induced migration and invasion in human PCa cells, MMP-2 inhibitors and siRNA were applied. As shown in Fig. 3f–i, either MMP-2 inhibitor or siRNA (Additional file 1: Figure S2A and B) abolishes the TSP-2-induced migration and invasion in PC-3 and DU145 cells. These results revealed that the TSP-2-augmented migration and invasion in PCa cells are MMP-2 dependent.

### The CD36 and integrin α_v_β_3_ are involved in the TSP-2-induced MMP-2 activation and the subsequent cell motility in human PCa cells

**Fig. 4:**
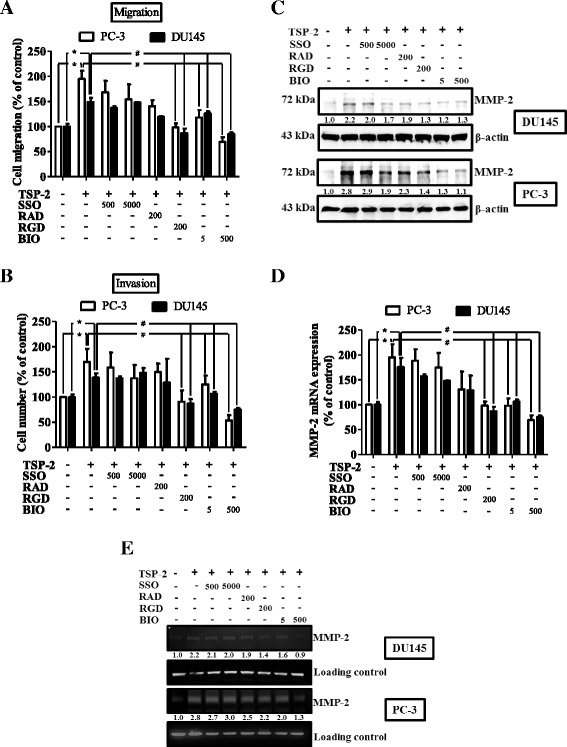
TSP-2-induced MMP-2 activation and migration were mediated through CD36 and integrin α_v_β_3_ in human PCa cells. Starved PC-3 (*open bar*) and DU145 (*closed bar*) cells were pretreated with SSO (500 and 5000 nM), RAD control peptide (200 nM), RGD peptide (200 nM), and BIO1211 (5 and 500 nM) for 1 h, followed by incubation of TSP-2 for another 24 h. The abilities of migration (**a**), invasion (**b**), as well as the expressions of MMP-2 protein (**c**), mRNA (**d**), and its activity (**e**) were assessed by the transwell assay, immunoblotting, real-time PCR, and zymography, respectively. Cells without treatments were used as a control (set to 100), and data were shown as multiples of that. Results are shown as the mean ± SEM (*n* ≥ 3, *p < 0.05 when compared to untreated control; ^#^ *p* < 0.05 when compared to the TSP-2-treated group)

Several membranous receptors, including CD36 [30], integrin α_4_β_1_ [31], and integrin α_V_β_3_ [32], have been shown to mediate the TSP-2’s physiological roles on cancers. To investigate which receptors are responsible for mediating the TSP-2-induced MMP-2 activation and migration in human PCa cells, chemical inhibitors for those receptors were applied (Additional file 1: Figure S4A and B). As shown in Fig. 4a, b, the TSP-2-induced migration and invasion were significantly inhibited upon BIO1221 and arginine-glycine-aspartic acid (RGD) peptide, but not by arginine-alanine-aspartic acid (RAD) control peptide or sulfo-*N*-succinimidyl oleate (SSO) treatments. This indicated that the TSP-2-induced migration and invasion is mediated through CD36 and integrin α_v_β_3_, but not by integrin α_4_β_1_. Moreover, inhibition of CD36 showed the higher suppressing effects on TSP-2-augmented cell motility than that by integrin α_v_β_3_. Meanwhile, these phenomena are similar to TSP-2’s effects on MMP-2 mRNA, protein, and activity in human PCa cells (Fig. 4c–e). These results suggested that the TSP-2-induced MMP-2 activation, cell migration, and invasion are mainly mediated through CD36, and partially through integrin α_V_β_3_, but not integrin α_4_β_1_ in human PCa cells.

### TSP-2 promotes cell migration and MMP-2 expression via the mitogen-activated protein kinase (MAPK) pathway in human PCa cells

**Fig. 5:**
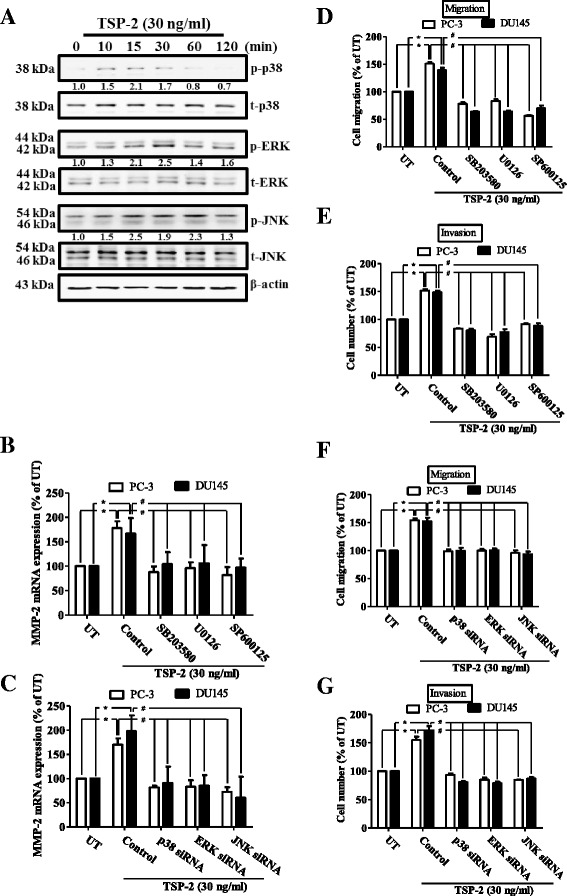
TSP-2-induced migration was mediated through the MAPK-dependent pathway in human PCa cells. **a** Starved DU145 cells were incubated with TSP-2 for 10, 15, 30, 60, and 120 min, and the phosphorylation of p38, ERK, and JNK were examined by immunoblotting using anti-phospho p38, ERK, and JNK antibodies, and followed by re-probing against to total p38, ERK, JNK, and β-actin to show equal loading amounts. The DU145 cells were pretreated with the p38 inhibitor (SB203580), ERK inhibitor (U0126), and JNK inhibitor (SP600125) for 1 h (**b**, **d**, and **e**), transfected with p38, ERK, and JNK siRNA (**c**, **f**, and **g**), and stimulated with TSP-2 for 24 h. The MMP-2 mRNA expression was measured by real-time PCR (**b** and **c**), and the migratory (**d** and **f**) and invasive (**e** and **g**) abilities were assessed by the transwell analysis. Cells without treatments were used as a control (set to 100), and data were shown as multiples of that. Results are shown as the mean ± SEM (*n* ≥ 3, *p < 0.05 when compared to untreated control; ^#^ *p* < 0.05 when compared to the TSP-2-treated group). *UT* untreated control

The MAPK is involved in several key signaling components and phosphorylation events that play important role in tumorigenesis, including migration and invasion [33]. To determine whether the MAPK pathway participates in TSP-2-induced migration on PCa cells, p38, ERK, and JNK signaling pathways were assessed. As shown in Fig. 5a, either p38, ERK, or JNK were phosphorylated following TSP-2 stimulation. To further study whether these MAPK signaling pathways were involved in TSP-2-induced MMP-2 expression and migration, MAPK inhibitors and siRNA were used. The mRNA and protein expressions of p38, ERK, and JNK were abolished upon their specific siRNA transfection (Additional file 1: Figure S2C and D). All these siRNAs showed no inhibitory effects on cell migration and invasion (Additional file 1: Figure S4C and D). Results showed that the TSP-2-induced MMP-2 mRNA expression, migration, and invasion were abolished upon inhibition of p38, ERK, and JNK pathways by using inhibitors or siRNA (Fig. 5b–g). These results demonstrated that TSP-2 induces cell migration and MMP-2 expression through the MAPK-dependent pathway.

### TSP-2 promotes cell migration and MMP-2 expression through down-regulation of microRNA-376c in human PCa cells

**Fig. 6:**
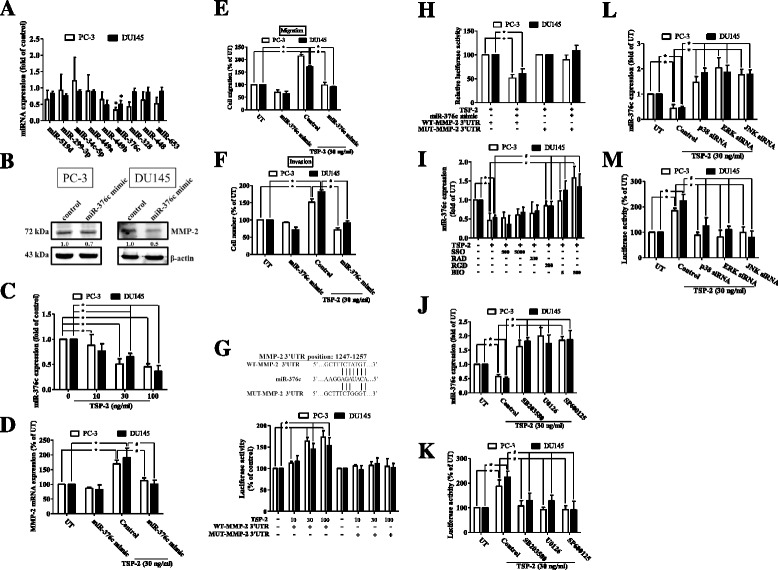
TSP-2 promotes migration and MMP-2 expression by down-regulating microRNA-376c expression in human PCa cells. **a** Starved PC-3 and DU145 cells were treated with or without 30 ng/ml of TSP-2 for 24 h, and the expressions of nine miRNAs which targets to MMP-2 3′UTR were measured by real-time PCR in PC-3 (*open bar*) and DU145 (*closed bar*) cells. Results were shown as multiple of untreated control. **b** PC-3 and DU145 cells were transfected with miR-376c mimic, and the MMP-2 protein expression was then determined by immunoblotting assay. **c** Starved PC-3 and DU145 cells were treated with 10, 30, and 100 ng/ml of TSP-2 for 24 h, and miR376c expression was then assessed by real-time PCR. The PC-3 and DU145 cells were transfected with miR-376c mimic, followed by treatment with TSP-2 for 24 h. MMP-2 mRNA expression (**d**), as well as the abilities of migration (**e**) and invasion (**f**) were then assessed. **g** PC-3 and DU145 cells were transfected with wild-type or mutant 3′UTR plasmid of MMP-2 containing a miR-376c binding site (*upper row*). The relative luciferase/renilla activities were then measured following 10, 30, and 100 ng/ml of TSP-2 stimulation. **h** PC-3 and DU145 cells were co-transfected with miR-376c and plasmids harboring with wild-type or mutant 3′UTR of MMP-2, followed by stimulation of TSP-2 for 24 h. The relative luciferase/renilla activities were then measured. Starved PC-3 and DU145 were pretreated with (**i**) SSO (500 and 5000 nM), RAD control peptide (200 nM), RGD peptide (200 nM), and BIO1211 (5 and 500 nM), or (**j** and **k**) SB203580, U0126, and SP600125 (2 μM) for 1 h, or (**l** and **m**) transfected with p38, ERK, and JNK siRNA, followed by incubation of TSP-2 for another 24 h. The miR-376c expression and the relative luciferase/renilla activities were then assessed. Cells without treatments were used as a control (set to 1 or 100), and data were shown as multiples of that. Results are shown as the mean ± SEM (*n* ≥ 3, *p < 0.05 when compared to untreated control; ^#^ *p* < 0.05 when compared to the TSP-2-treated group). *UT* untreated control, *WT* wild type, *MUT* mutant

Previous studies have shown that microRNA (miRNA) plays important roles on regulation of cancer progression and metastasis [20]. To investigate whether miRNA is responsible for the TSP-2-induced MMP-2 expression, nine predicted miRNAs that contain binding sites of the 3′UTR of MMP-2, identified by microRNA.org (http://www.microrna.org/microrna/home.do), were studied. Results showed that the expression of miR-376c was mostly down-regulated following TSP-2 stimulation (Fig. 6a) and via a dose-dependent manner (Fig. 6c and Additional file 1: Figure S1C) in PC-3, DU145, and LNCaP cells. To validate whether MMP-2 serves as a target for miR-376c, MMP-2 protein expression was monitored following miR-376 mimic transfection. Results showed that MMP-2 protein expression was obviously decreased upon miR-376 mimic transfection in DU145 and PC-3 cells (Fig. 6b), indicating that MMP-2 is a miR-376c target. To illustrate whether miR-376c is involved in the TSP-2-induced MMP-2 expression and migration, the synthesized miR-376c mimic was applied. Results showed that the TSP-2-induced MMP-2 mRNA expression, migration, and invasion were all abolished upon miR-376c mimic transfection in PC-3 and DU145 cells (Fig. 6d–f). To further confirm whether miR-376c was specifically targeted to MMP-2, the luciferase reporter vectors harboring wild-type or mutant 3′UTR of MMP-2 were constructed (Fig. 6g). These vectors were transfected into PC-3 and DU145 cells followed by treatment with TSP-2, and the luciferase activity was then assessed. The luciferase activity was increased only in wild-type MMP-2 3′UTR transfection, but not in mutant MMP-2 3′UTR, and via a concentration-dependent manner (Fig. 6g). Meanwhile, the TSP-2-augmented MMP-2 luciferase activity was attenuated upon miR-376c mimic transfection (Fig. 6h), indicating that the TSP-2-induced MMP-2 expression is mediated by down-regulating miR-376c expression.

To decipher the molecular mechanism of TSP-2’s effects on miR-376c, inhibitors of the membranous receptors of TSP-2 were applied. Results showed that the TSP-2-decreased miR-376c expression was reversed upon the BIO1211 and RGD peptide, but not by SSO stimulation (Fig. 6i), indicating that the receptors of CD36 and integrin α_V_β_3_, but not integrin α_4_β_1_, are responsible for the TSP-2-down-regulated miR-376c expression. To determine the intracellular signaling of the TSP-2-down-regulated miR-376c, the MAPK pathways were studied. Results showed that the TSP-2-reduced miR-376c expression was rescued by treatment with p38, ERK, or JNK inhibitors (Fig. 6j) or their siRNAs (Fig. 6l). Interestingly, the miR-376c expression is sensitive to the inhibition of p38, ERK, and JNK (Additional file 1: Figure S4E and F), especially in the presence of TSP-2 (Fig. 6j, l), suggesting that some TSP-2-independent pathway may play roles on miR-376c regulation. Meanwhile, the TSP-2-induced miR-376c binding ability was also suppressed upon knockdown of p38, ERK, and JNK signaling pathways (Fig. 6k, m). These results indicated that miR-376c was down-regulated by TSP-2 via the CD36, integrin α_v_β_3_, and MAPK-dependent pathway in human PCa cells.

### Knockdown of TSP-2 decreases PCa cell migration and osteolytic metastasis in vivo

**Fig. 7:**
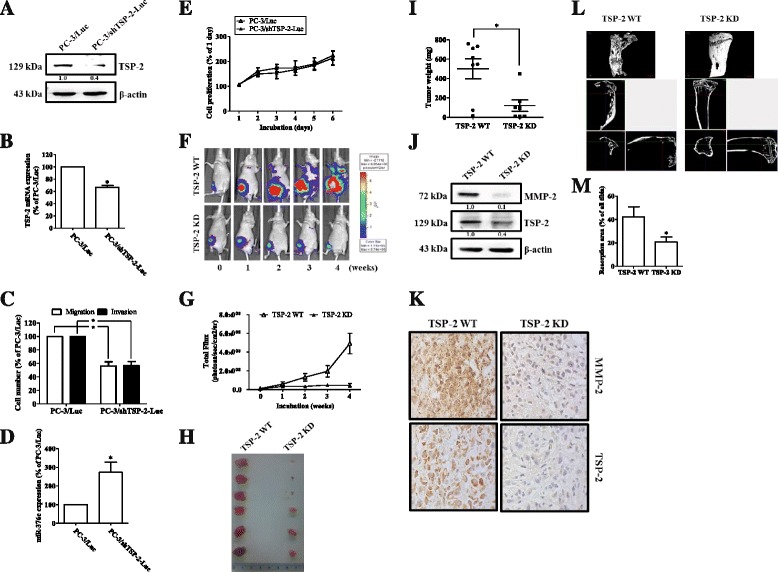
Knockdown of TSP-2 decreases cell migration and osteolytic metastasis in vivo. PC-3 constitutively expressed pLenti CMV V5-Luc cells were a transfected scramble of (PC-3/Luc) or TSP-2 shRNA (PC-3/shTSP-2-Luc), followed by determining their TSP-2 protein (**a**), or mRNA (**b**), and miR-376c (**d**) expressions by immunoblotting and real-time PCR, respectively. The migration (*open bar*), invasion (*closed bar*) (**c**), or cell viability (**e**) were also determined by the transwell assay and MTT analysis, respectively. **f** These cells were injected into the right tibia bone of nude mice and detection by bioluminescence imaging at the indicated time intervals, and (**g**) these images were quantified (photons/s of the right leg). After 4 weeks, these mice were sacrificed, and the tumors were excised (**h**) and their weights were determined (**i**). The expressions of TSP-2 and MMP-2 on these tumors were then assessed by immunoblotting (**j**) or IHC staining (**k**). **l** Represents the micro-CT images of the tibia from sacrifice mice. Scale bar = 1 mm. **m** This represents quantification of the resorption area of the tibia. *WT* wild type, *KD* knockdown

To confirm the role of TSP-2 in PCa metastasis in vivo, we took advantage of PC-3 cells that stably express pLenti CMV V5-Luc (PC-3/Luc) and transfected with TSP-2 shRNAs. Results showed that the protein and mRNA expressions of TSP-2 were significantly abolished in stably expressing TSP-2 shRNA PC-3 cells (PC-3/shTSP-2-Luc) (Fig. 7a, b). In addition, the migratory and invasive abilities of PC-3/shTSP-2-Luc cells were obviously decreased compared to that in control cells (PC-3/Luc) (Fig. 7c). Furthermore, the expression of miR-376c in PC-3/shTSP-2-Luc cells was higher than that in PC-3/Luc cells (Fig. 7d), indicating that miR-376c expression is negatively correlated to TSP-2. Moreover, the proliferation rate between PC-3/Luc and PC-3/shTSP-2-Luc cells was almost same (Fig. 7e), indicating that TSP-2 knockdown did not affect cell proliferation rate. Previous studies have indicated the critical roles of MMP-2 in bone metastasis in various tumors. MMP-2 expression is contributed to matrix degradation and osteolytic bone metastasis in many tumors such as prostate cancer [34], breast cancer [35, 36], and renal cell carcinoma [37]. To explore the role of TSP-2/MMP-2 axis in bone metastasis of prostate cancer, we performed mouse models of bone metastasis by intratibial injection of cancer cells, and the bone metastasis was then detected by bioluminescence imaging. Our results showed that knockdown of TSP-2 significantly inhibited PCa metastasis in vivo (Fig. 7f, g). Moreover, the TSP-2 expression is positively associated with MMP-2 expression in vivo, as demonstrated by immunoblotting and IHC staining (Fig. 7j, k), indicating that TSP-2 decreases MMP-2 expression and the subsequent metastasis in vivo. On the other hand, the metastatic tumor growth and osteolytic area were significantly reduced in the TSP-2 knockdown group (Fig. 7h, i, l, and m), suggesting that TSP-2 is essential for tumor invasion in vivo. Taken together, the inhibition of TSP-2 on PCa suppresses bone metastasis in vivo.

### The expressions of TSP-2 and MMP-2 are correlated with the Gleason score of PCa

**Fig. 8:**
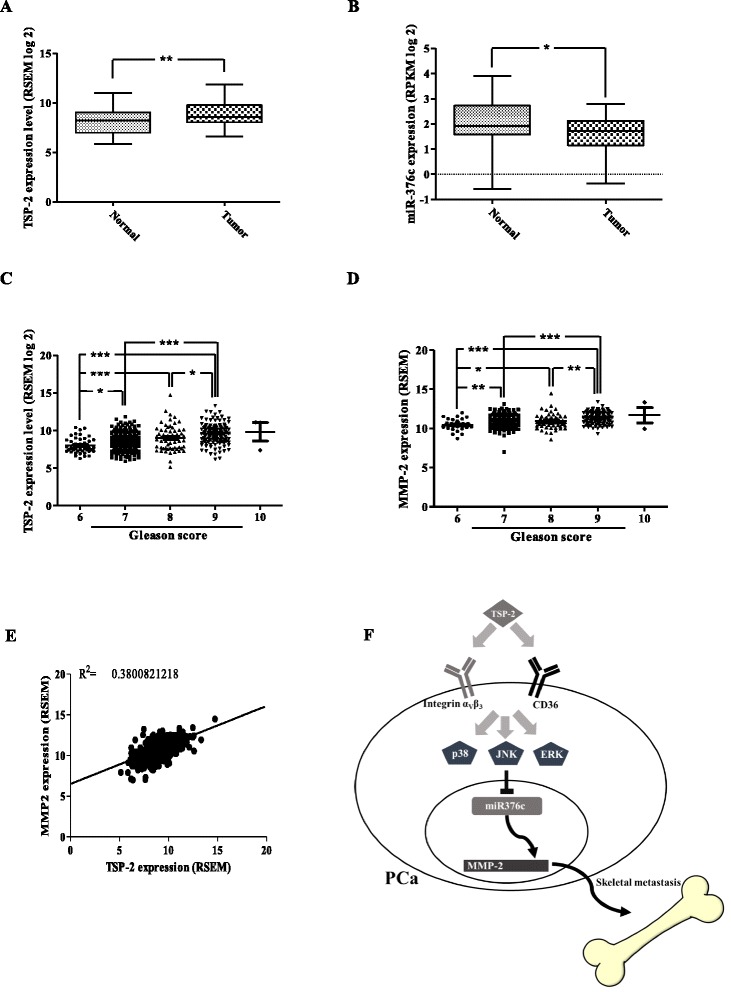
The expression of TSP-2 is correlated with Gleason score of PCa and MMP-2 expression. The expression of TSP-2 (**a**) and miR-376c (**b**) between healthy individuals and PCa patients was determined from 52 cases of The Cancer Genome Atlas (TCGA) database. The correlation of TSP-2 (**c**) and MMP-2 (**d**) with PCa progression was determined from 45, 245, 63, 136, and 3 individuals of PCa patients from the Gleason score 6~10 of TCGA database. The one-way ANOVA with Bonferroni’s multiple comparisons test was performed, and *p* ≤ 0.05 showed significance. **e** The correlation gene expression between TSP-2 and MMP-2 was illustrated from 493 cases of TCGA database. **f** The working model represents the roles of TSP-2 on skeletal metastasis of PCa

To further verify the roles of TSP-2 on PCa progression, the correlation of TSP-2 and PCa progression was determined by The Cancer Genome Atlas (TCGA) database. Results indicated that PCa patients show the higher TSP-2 expression level than those on health individuals (Fig. 8a). Furthermore, the expression levels of TSP-2 and MMP-2 were increased with the Gleason score of PCa (Fig. 8c, d). Meanwhile, the expression of TSP-2 is positively correlated with MMP-2 (Fig. 8e). However, the expression of miR-376c showed the slightly decreased in PCa tumor (Fig. 8b). In summary, these results suggested that TSP-2 plays a positive role on PCa progression.

## Discussion

TSP-2 has been demonstrated to play an anti-angiogenic role on tumor cells [11, 15]. However, its expression profile is controversial, being down-regulated in some tumors [9, 16], while being overexpressed in others [17–19]. Thus, the biological functions of TSP-2 are still uncertain, especially in PCa. Our IHC results indicated that TSP-2 expression was positively correlated with PCa progression, and this result is consistent with the TSP-2 expression pattern in the other datasets of PCa. Moreover, the TCGA dataset of PCa consolidates our finding in this study. The TSP-2 expression is higher in PCa patients and correlated with tumor progression.

TSP-2 exhibit an extensive modular domains named exosites, which have been implicated in interaction with various cell surface, matrix, and proteolytic proteins. Moreover, the properdin-like type 1 repeats (TSR) of TSP-2 were found to interact with MMP-2 and endocytosis of complexes by LRP. This mechanism reveals the modulation of MMP-2 secretion from cells by TSP-2 expression [14]. Although TSP-2 binds to MMP-2 that regulates the extracellular levels of MMP-2 during collagen fibrillogenesis, wound healing, and angiogenesis [13], little is known about the roles of TSP-2 on PCa progression. To investigate the biological roles of TSP-2, PCa cell lines, PC-3, DU145, and LNCaP were applied. We revealed that TSP-2 induces MMP-2 expression and the subsequent migration and invasion in PC-3, DU145, and LNCaP cells. Moreover, the migratory ability of PC-3 is significantly higher than that in DU145 cells. This result is consistent to other studies showing the higher motility in PC-3 cells [38]. It may have resulted from the higher expression of TSP-2 in PC-3, thus inducing more MMP-2 expression. Furthermore, the higher TSP-2 expression level showed the higher MMP-2 expression, migratory, and invasive abilities which were also observed in PC-3-I3 and DU145-I5, compared to PC-3 and DU145, respectively. Meanwhile, the knockdown of TSP-2 also suppressed the migratory and invasive abilities in PC-3 cells through attenuating MMP-2 expression. These results confirmed the biological roles of TSP-2 in human PCa cells. Furthermore, we also showed that metastatic PCa has a higher TSP-2 and MMP-2 expression when compared with normal prostate tissue, suggesting that TSP-2 might be associated with advanced PCa. Moreover, bone metastasis is obviously inhibited while TSP-2 is knocked down when compared to wild-type mice in vivo. Taken together, these results reveal that the TSP-2 expression level is associated with tumor metastasis, suggesting that TSP-2 could be used as a biomarker of PCa progression.

The development of an antiangiogenic target has been a common strategy for cancer therapy for a long time, but substantial benefits remain unrealized because tumors elicit evasive resistance [39]. Interestingly, the inhibition of VEGFR/PDGFR by applying their kinase inhibitors sunitinib/SU11248 can accelerate metastatic tumor growth and decrease overall survival in mice, but cannot function as an anti-cancer strategy [40]. TSP-1 and TSP-2 were considered as an anti-cancer molecular by inhibiting angiogenesis through antagonizing VEGF expression or suppressing metastasis by manipulating MMPs activity in several cancers, including PCa [41, 42]. Surprisingly, under hypoxia conditions, TSP-1 might trigger cell migration in advanced PCa cells [43], suggesting that the anti-angiogenic molecules may switch to play a positive role for tumor cell migration. Herein, we showed for the first time that the expression TSP-2 is correlated to the PCa progression, especially on the metastasis. Since, TSP-2 is the homology to TSP-1, we considered that TSP-2 may share the similar mechanism to TSP-1, showing the promotion of the migratory effects on tumor progression. Further experiments are needed for clarifying this issue in the future.

TSP-1 and TSP-2 have been known as the anti-angiogenic molecules which may bind to CD36 [30, 41]. Interestingly, the inhibition between TSP-1 and CD36 by the TSP1 antibody A4.1 blocks the TSP-1’s effects on anti-angiogenesis [44] and also suppresses the migration of C4-2 cells [43], suggesting that the CD36 is responsible for the TSP-1-induced migration. This result is similar to our study showing that CD36 participated in the TSP-2-induced MMP-2 expression and the subsequent migration and invasion. In addition to CD36, TSP-2 also regulates angiogenesis and cell motility, through integrins, including integrin α_4_β_1_ [31] and α_V_β_3_ [32]. Integrins are a large family of cell-surface glycoproteins, which bind to several extracellular matrix components and regulate cytoskeletal organization and facilitate cell motility. Overexpression of integrin α_V_β_3_ has been found in many cancers, including melanoma, prostate, and breast cancer and is associated with their malignancy and responsible for their bone metastasis [45, 46]. Herein, we also showed for the first time that the TSP-2’s effects on PCa cell migration and invasion are mediated through integrin α_V_β_3_, but not integrin α_4_β_1_ in human PCa cells.

It has been shown that miRNAs are involved in multiple biological processes and are also tightly correlated with tumor progression, including PCa [20]. The miR-376c belongs to the miR-376 cluster gene family, containing miR-376a, miR-376a*, and miR-376b. The lower miR-376c level is correlated with a higher PSA and Gleason score [10], suggesting that miR-376c is a negative regulator for PCa progression. Furthermore, miR-376c is shown to serve as an important regulator for androgen signaling by targeting the 3′UTR of UDP-glucuronosyltransferase 2B15 and UGT2B17 in PCa cells [47]. These studies are similar to our results showing that the miR-376c expression level is negatively associated with cell migration, invasion, and PCa progression. Meanwhile, we also showed the first evidence that miR-376c is essential for the TSP-2-induced migration in PCa cells. Thus, the modulation of miR-376c expression may develop as a novel strategy for PCa therapy.

To understand the physiological role of TSP-2 in vivo, we established a PC-3 cells with stably knockdown of its TSP-2 (PC-3/shTSP-2-Luc). We found that the expression of MMP-2, as well as the abilities of migration and invasion was significantly reduced in PC-3/shTSP-2-Luc cells, whereas its miR-376c is higher than normal PC-3/Luc cells. Through monitoring by bioluminescence imaging, we showed that the knockdown of TSP-2 dramatically suppresses bone metastasis and osteolytic abilities of prostate tumor. Interestingly, the tumor with PC-3/shTSP-2-Luc is smaller than that with PC-3/Luc, although the cell proliferation rates between these cells are almost the same. We considered that TSP-2 might regulate osteoclastogenesis and bone remodeling in vivo. This issue needs further investigation in the future.

The concept of “vicious cycle” has been well established in bone metastasis. The tumor-bone interaction promotes both bone destruction and tumor growth during bone metastasis [48]. In the past, the role of MMPs in tumor progression is focused on invasion and metastasis. However, the crucial role of MMPs in vicious cycle of bone metastasis has been discussed recently. MMPs improve the tumor growth not only through bone destruction but also by regulating bioactivity of the vicious cycle-related factors such as PTHrP, RANKL, and TGFβ [49]. Here, our findings reveal that TSP-2/MMP-2 axis in bone metastasis may be regulated through complicated mechanism and remains to be discussed in the future.

## Conclusions

In recent years, numerous studies have been addressed to show the role of TSP-2 in tumor progression as an anti-angiogenesis by inhibiting cell migration. However, the role of TSP-2 in PCa has not been examined in detail. Here, we showed for the first time that TSP-2 plays a positive role in PCa progression. It promotes cell migration in vitro and in vivo. We also showed that TSP-2 promotes metastasis by down-regulation of miR-376c through the MAPK signaling pathway (Fig. 8f). This result demonstrates that TSP-2 may represent a promising new target for treating PCa.

## Additional file

Additional file 1: Figure S1. TSP-2 induces MMP-2 and inhibits miR-376c expressionsin LNCaP cells. **Figure S2.** The siRNAs abolished the target genes in PC-3 cells. **Figure S3.** TSP-2 promotes cell migratory and invasive abilities in PC-3 and DU145 cells. **Figure S4.** The cell migration, invasion and miR-376c expression were not regulated by chemical inhibitor stimulation in PC-3 and DU145 cells. (PDF 1140 kb)

## Abbreviations

3′UTR: The 3′ untranslated region
IHC: Immunohistochemistry
IVIS: The in vivo imaging system
miRNA: MicroRNA
MMP: Matrix metalloproteinase
PCa: Prostate cancer
siRNA: Small interfering RNA
TSP-2: Thrombospondin-2
