# Supplementary material for: Thrombospondin-2 promotes prostate cancer bone metastasis by the up-regulation of matrix metalloproteinase-2 through down-regulating miR-376c expression
Source: J Hematol Oncol. 2017 Jan 25;10:33. doi: 10.1186/s13045-017-0390-6 (PMC5264454; doi:10.1186/s13045-017-0390-6)
Supplement: Additional file 1: Figure S1. — TSP-2 induces MMP-2 and inhibits miR-376c expressionsin LNCaP cells. Figure S2. The siRNAs abolished the target genes in PC-3 cells. Figure S3. TSP-2 promotes cell migratory and invasive abilities in PC-3 and DU145 cells. Figure S4. The cell migration, invasion and miR-376c expression were not regulated by chemical inhibitor stimulation in PC-3 and DU145 cells. (PDF 1140 kb) [file 13045_2017_390_MOESM1_ESM.pdf]

Supplementary Results

TSP-2 induces MMP-2 expression in LNCaP cells

To investigate the roles of TSP-2 on LNCaP cells, 10, 30 and 100 ng/ml of TSP-2 were applied. Results showed that both MMP-2 mRNA and protein were increased upon TSP-2 stimulation via a concentration-dependent manner (Supplementary Figure 1A and 1B). To further confirm whether TSP-2 also suppresses miR-376c expression in LNCaP cells, real-time PCR was applied. Results revealed that 30 and 100 ng/ml of TSP-2 suppressed significantly miR-376c expression in LNCaP cells (Supplementary Figure 1C). Taken together, these results indicated that the TSP-2-induced MMP-2 expression also observed in the androgen-independent LNCaP cells.

The siRNAs specifically abolished the target gene expression in PC-3 cells

To clarify whether MMP-2 is involved in the TSP-2-induced migration and invasion in human PCa cells, MMP-2 siRNA were applied. Results showed that the MMP-2 mRNA and protein expression were suppressed 61.4% and 59.6%, respectively, upon MMP-2 siRNA transfection in PC-3 cells (Supplementary Figure 2A and 2B). To confirm the MAPK signaling pathways is responsible for the TSP-2-induced MMP-2, migration, and invasion in PC-3 cells, p38, ERK, and JNK siRNAs were applied. Results showed that the mRNA and protein expressions of p38, ERK and JNK were

specifically knockdown by their siRNA (Supplementary Figure 2C and 2D).

TSP-2 induces migration and invasion in PC-3 and DU145 cells

To investigate the migratory effect of TSP-2 on PCa cells, PC-3 and DU145 cells were treated with various concentrations of TSP-2. Results showed that both migratory and invasive activities significantly increased upon TSP-2 treatment via a concentration-dependent manner (Supplementary Figure 3A). To further confirm the roles of TSP-2 on PC-3 and DU145 cells, the PC-3-I3 and DU145-I5 invasive prostate cancer cells were established in the transwell plate after 3 and 5 rounds of selection, respectively. Results indicated that PC-3-I3 and DU145-I5 showed the higher migratory and invasive abilities than PC-3 and DU145 cells, respectively (Supplementary Figure 3B).

The chemical inhibitors and siRNAs showed no effects on cell migration, invasion and miR-376c expression

To investigate whether the inhibitors of CD36, integrin $\alpha_4\beta_1$, and integrin $\alpha_V\beta_3$ possess the inhibitory effects on cell migration and invasion in absence of TSP-2, these inhibitors were applied. Results showed that the abilities of cell migration and invasion were not inhibited by all of the inhibitors stimulation in PC-3 and DU145 cells

(Supplementary Figure 4A and 4B). To clarify inhibitions of p38, ERK, and JNK affect the cell migration, invasion and miR-376c expression on PCa cells, these inhibitors were applied. Results revealed that the inhibitors of p38, ERK and JNK showed no effects on cell migration and invasion abilities in PC-3 and DU145 cells (Supplementary Figure 4C and 4D). Interestingly, miR-376c expression was slightly increased upon inhibition of p38, ERK or JNK in PC-3 and DU145 cells (Supplementary Figure 4E and 4F).

Supplementary Figure Legends

Supplementary Figure 1. TSP-2 induces MMP-2 and inhibits miR-376c expressions in LNCaP cells.

Starved LNCaP cell were incubation with TSP-2 (10-100 ng/ml) for 24 h, and the MMP-2 protein (A), mRNA (B) and miR-376c (C) expressions were analyzed through immunoblotting and real-time PCR, respectively. Cells without treatments were used as a control (set to 100), and data were shown as multiples of that. Results are shown as the mean±SEM (n≧3). * $p < 0.05$ when compared to untreated control.

Supplementary Figure 2. The siRNAs abolished the target genes in PC-3 cells.

PC-3 cells were transfected with siRNAs of scramble, MMP-2 (A and B), p38, ERK or JNK (C and D) for 24 h, and the knockdown efficiencies were then assessed by immunoblotting (B and D) or real-time PCR (A and B). Cells transfected with scramble siRNA were used as a control (set to 1.0), and data were shown as multiples of that. * $p < 0.05$ when compared to scramble siRNA-transfected control.

Supplementary Figure 3. TSP-2 promotes cell migratory and invasive abilities in PC-3 and DU145 cells.

(A) PC-3 and DU145 cells were incubation with TSP-2 (10-100 ng/ml) for 24 h,

and their abilities of migration and invasion were measured by the transwell assay. (B) The highly migratory PC-3-I3 and DU145-I5 cells were selected by the transwell plate for 3 or 5 rounds of selection, respectively. The migration and invasion of these cells were then conformed by the transwell assay.

Supplementary Figure 4. The cell migration, invasion and miR-376c expression were not regulated by chemical inhibitor stimulation in PC-3 and DU145 cells.

PC-3 (open bar) and DU145 (closed bar) cells were treated with vehicle control or (A and B) SSO (500 and 5000 nM), RAD control peptide (200 nM), RGD peptide (200 nM), BIO1211 (5 and 500 nM), or (C and D) 2 μM of SB203580, U0126 and SP600125 for 24 h, and their abilities of migration (A and C) and invasion (B and D) were measured by the transwell assay. PC-3 (open bar) and DU145 (closed bar) cells were treated with vehicle control, 2 μM of SB203580, U0126 and SP600125 (E), or transfected with siRNAs of scramble control, p38, ERK and JNK (F) for 24 h, miR376c expression was then assessed by real-time PCR. Cells treated with vehicle control or transfected with scramble siRNA were used as a control (set to 100 or 1.0, respectively), and data were shown as multiples of that.

Figure S1

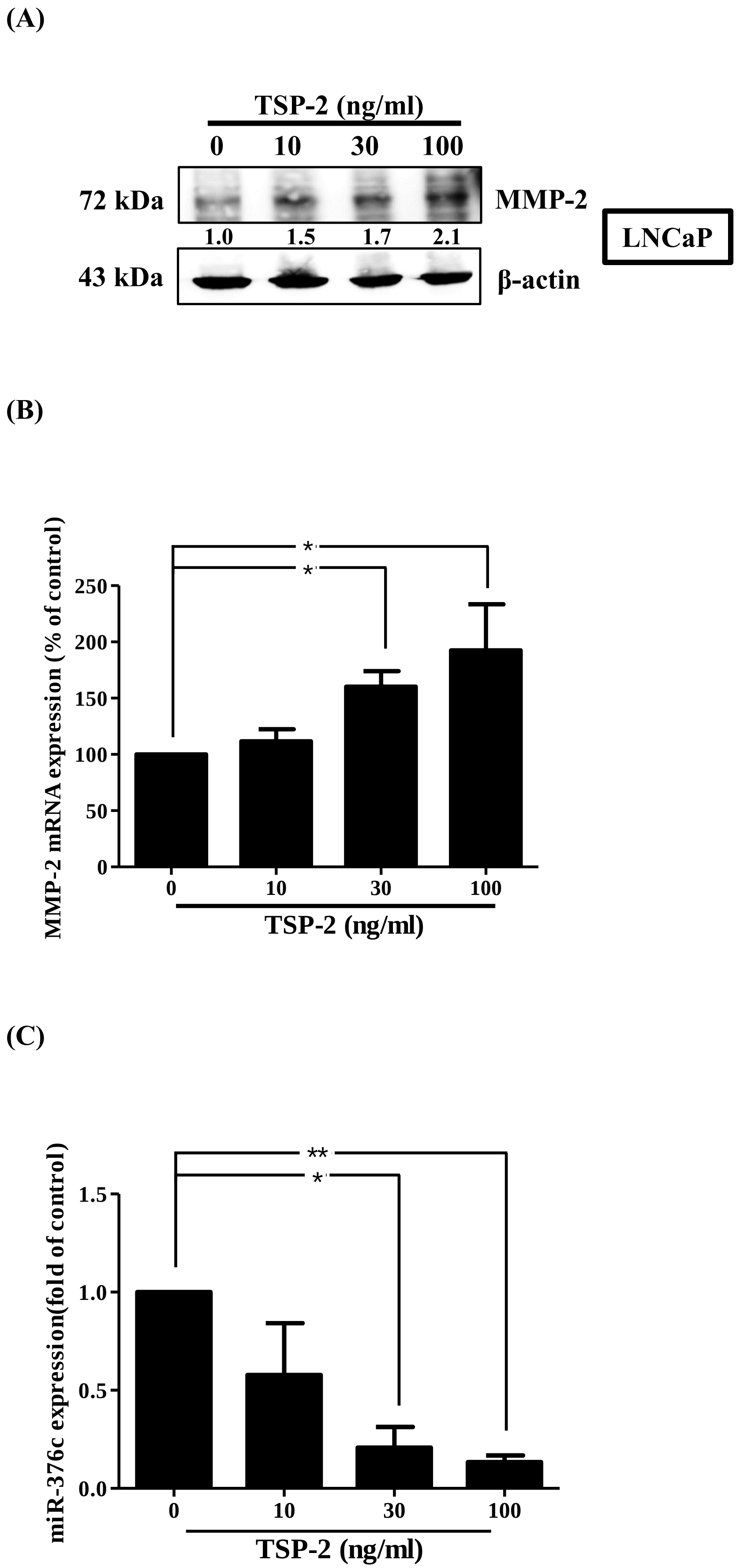
(A)
TSP-2 (ng/ml)
0
10
30
100
72 kDa
MMP-2
1.0
1.5
1.7
2.1
43 kDa
β-actin
LNCaP
(B)
MMP-2 mRNA expression (% of control)
*
*
250
200
150
100
50
0
0
10
30
100
TSP-2 (ng/ml)
(C)
miR-376c expression(fold of control)
**
*
1.5
1.0
0.5
0.0
0
10
30
100
TSP-2 (ng/ml)

Figure S2

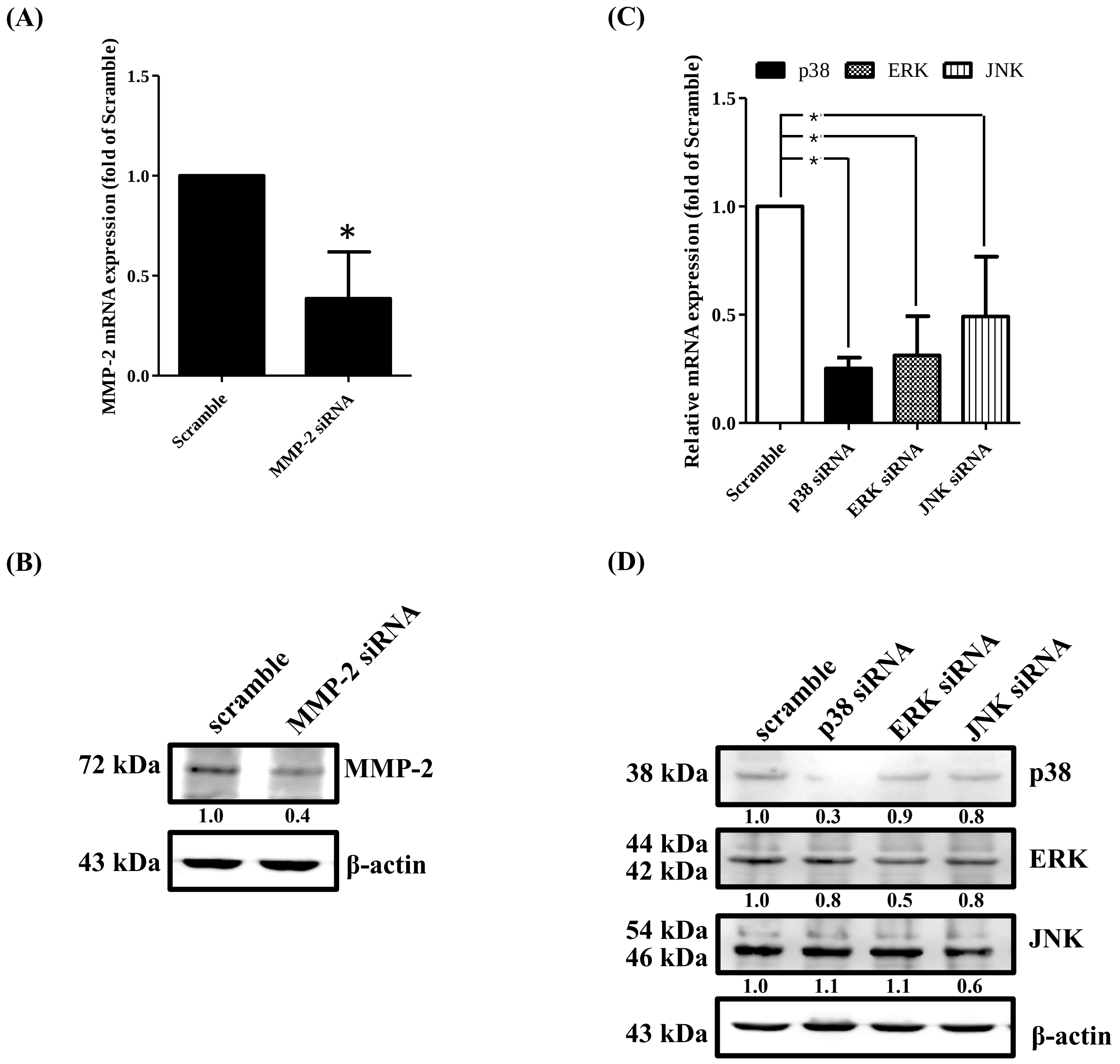
(A)
MMP-2 mRNA expression (fold of Scramble)
1.5
1.0
0.5
0.0
*
Scramble
MMP-2 siRNA
(C)
p38
ERK
JNK
Relative mRNA expression (fold of Scramble)
1.5
1.0
0.5
0.0
*
*
*
Scramble
p38 siRNA
ERK siRNA
JNK siRNA
(B)
scramble
MMP-2 siRNA
72 kDa
MMP-2
1.0
0.4
43 kDa
β-actin
(D)
scramble
p38 siRNA
ERK siRNA
JNK siRNA
38 kDa
p38
1.0
0.3
0.9
0.8
44 kDa
42 kDa
ERK
1.0
0.8
0.5
0.8
54 kDa
46 kDa
JNK
1.0
1.1
1.1
0.6
43 kDa
β-actin

(A)

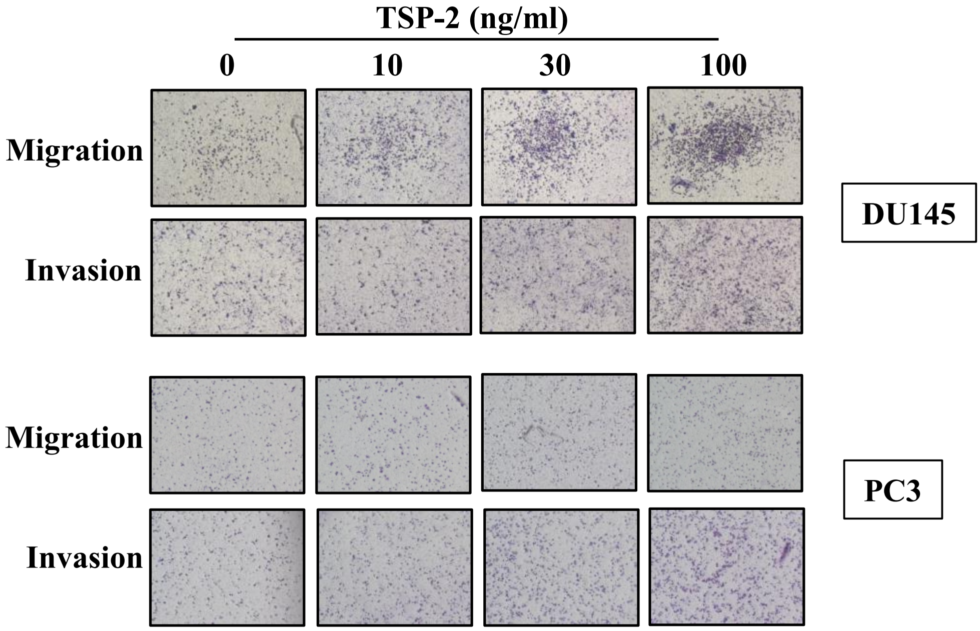
TSP-2 (ng/ml)
0
10
30
100
Migration
Invasion
DU145
Migration
Invasion
PC3

(B)

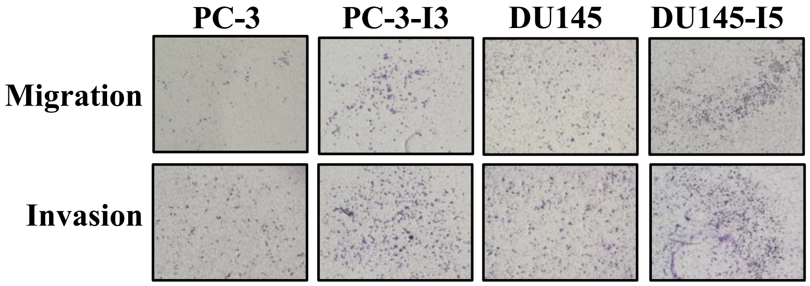
PC-3
PC-3-I3
DU145
DU145-I5
Migration
Invasion

Figure S4

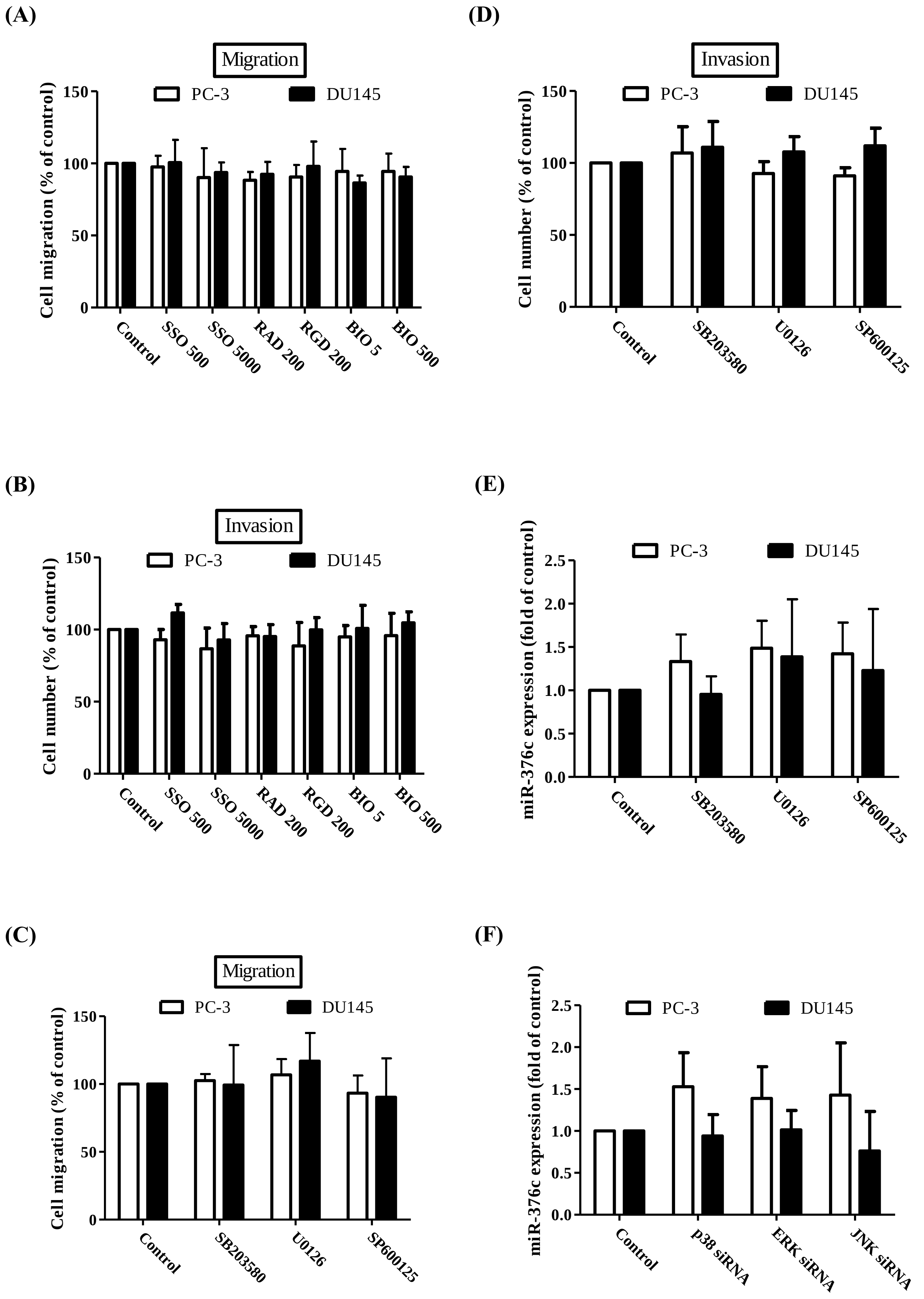

(A)
Migration
PC-3
DU145
Cell migration (% of control)
150
100
50
0
Control
SSO 500
SSO 5000
RAD 200
RGD 200
BIO 5
BIO 500
(D)
Invasion
PC-3
DU145
Cell number (% of control)
150
100
50
0
Control
SB203580
U0126
SP600125
(B)
Invasion
PC-3
DU145
Cell number (% of control)
150
100
50
0
Control
SSO 500
SSO 5000
RAD 200
RGD 200
BIO 5
BIO 500
(E)
PC-3
DU145
miR-376c expression (fold of control)
2.5
2.0
1.5
1.0
0.5
0.0
Control
SB203580
U0126
SP600125
(C)
Migration
PC-3
DU145
Cell migration (% of control)
150
100
50
0
Control
SB203580
U0126
SP600125
(F)
PC-3
DU145
miR-376c expression (fold of control)
2.5
2.0
1.5
1.0
0.5
0.0
Control
p38 siRNA
ERK siRNA
JNK siRNA
